# A Comparative Study of 3D UE Positioning in 5G New Radio with a Single Station

**DOI:** 10.3390/s21041178

**Published:** 2021-02-08

**Authors:** Bo Sun, Bo Tan, Wenbo Wang, Elena Simona Lohan

**Affiliations:** Electrical Engineering Unit, Faculty of Information Technology and Communication Sciences, Tampere University, 33720 Tampere, Finland; bo.tan@tuni.fi (B.T.); wenbo.wang@tuni.fi (W.W.); elena-simona.lohan@tuni.fi (E.S.L.)

**Keywords:** positioning, uniform rectangular array (URA), joint time-angle delay estimation, expectation-maximization (EM), interference cancellation, 5G NR, reference signal

## Abstract

The 5G network is considered as the essential underpinning infrastructure of manned and unmanned autonomous machines, such as drones and vehicles. Besides aiming to achieve reliable and low-latency wireless connectivity, positioning is another function provided by the 5G network to support the autonomous machines as the coexistence with the Global Navigation Satellite System (GNSS) is typically supported on smart 5G devices. This paper is a pilot study of using 5G uplink physical layer channel sounding reference signals (SRSs) for 3D user equipment (UE) positioning. The 3D positioning capability is backed by the uniform rectangular array (URA) on the base station and by the multiple subcarrier nature of the SRS. In this work, the subspace-based joint angle-time estimation and statistics-based expectation-maximization (EM) algorithms are investigated with the 3D signal manifold to prove the feasibility of using SRSs for 3D positioning. The positioning performance of both algorithms is evaluated by estimation of the root mean squared error (RMSE) versus the varying signal-to-noise-ratio (SNR), the bandwidth, the antenna array configuration, and multipath scenarios. The simulation results show that the uplink SRS works well for 3D UE positioning with a single base station, by providing a flexible resolution and accuracy for diverse application scenarios with the support of the phased array and signal estimation algorithms at the base station.

## 1. Introduction

Accurate and robust positioning is becoming a core requirement for future autonomous vehicles. Location information will serve not only for seamless tracking of the autonomous vehicle in order to allow remote control and to avoid collisions, but also as an enabler for situation and context awareness [1], for improved communication functions [2,3], for optimized path planning [4], and for location-based authentication and enhanced security of communications [5].

Though any Global Navigation Satellite System (GNSS) is currently able to provide centimeter-level accuracy outdoors (e.g., with the help of professional multi-frequency GNSS receivers), it is well known that GNSS suffers from interferences, multipath, and a low signal-to-noise ratio in dense urban environments, where many of the future autonomous vehicles will be deployed (e.g., industrial drones, autonomous robots helping people who are blind, etc.). Complementary solutions to GNSS are necessary, and there are currently two options on the table, namely non-cellular systems (i.e., WiFi, Bluetooth Low Energy (BLE), Ultra Wide-Band (UWB), and Long-Range wireless networks (LoRa)) and cellular systems (i.e., GSM, 3G, LTE, and the emerging 5G systems). One obvious advantage of cellular-based localization techniques over the non-cellular ones is less interference in their frequency bands, as they typically use the licensed spectrum, unlike the non-cellular solutions typically operating in the unlicensed Industrial, Scientific, and Medical (ISM) bands, which are heavily polluted. Using cellular for positioning can be traced back to the GSM era. The typical methods used in the GSM system include the cell identification (CID)-based [6] and receiving signal strength-based (RSS) [7] approximating ones that inspired coarse location-based (∼550 m accuracy [8]) social media services. Later in the 3G network, the accuracy of the CID-based approach was improved because of the use of smaller cells. More importantly, the timing-based approaches (TDoA, OTDoA, round-trip time (RTT), etc.) pushed the positioning accuracy to the range between 50∼200 m [8]. Recent cellular systems such as 4G and 5G have, by design, signaling or reference signals dedicated to synchronization, channel estimation, and localization and therefore can offer competitive performance with respect to the GNSS also in dense urban scenarios. The typical signals for these purposes include the positioning reference signals (PRSs) in LTE Release-8 [9] and the CSI reference signal (CSI-RS) and sounding reference signal (SRS) [10] in 5G NR.

Amongst the existing cellular-based localization solutions, 5G new radio (NR) was born to meet the keen needs of high-precision positioning, which is an inevitable technique for many future vertical applications. To be specific, 5G NR allows multi-/single-cell positioning: (i) the sounding reference signal (SRS) is employed for the base station (BS) to perform uplink time difference of arrival (TDoA); (ii) the enhanced capability of beamforming, especially in the mmWave domain, enables the BS to measure the angle of arrival (AoA); (iii) both the BS and user equipment (UE) could apply round trip time (RTT) measurements, which have a low demand for time synchronization and provide extra flexibility for radio planning. In brief, 5G NR promises rich resources for a vast range of vertical applications in the near future. 5G positioning has been studied in many works. For example, a weighted centroid geometric (WCG) solution and an extended Kalman filter (EKF)-based positioning based on AoA and ToA measurements were described in [11]. Distinct from this work, the AoA and ToA initial measurements in [11] were based on Cramér–Rao lower bounds (CRLBs) rather than on estimated values, and the study in [11] focused on the multi-station case, and not on the single-station case as here. 5G positioning with mmWave signals was also studied in [12], with the focus on downlink configurations, not on uplink positioning. Furthermore, EKF was used in [12] to compute the joint UE position and base station orientation. Only the multi-station scenario, with four base stations, was studied in [12]. Another downlink 5G positioning method with joint ToA and AoA measurements was studied in [13] for a single base station. The positioning in [13] relied on a maximum likelihood estimator and on CRLBs instead of actual AoA and ToA estimates. AoA estimation through an ESPRIT algorithm was recently addressed in [14]. A generic mmWave multiple-input multiple-output (MIMO) signal was considered in [14], and the focus was only on azimuth angle estimation, not a joint azimuth-elevation estimation. Another work focusing on angle estimation in 5G mmWave systems was [15]. The focus there was also on azimuth elevation, and estimation errors below $2.8^{\circ }$ were achieved.

The focus of this work is the single-station 3D positioning of the UE in the sub-6 GHz band by using the radio signal propagation properties. The subspace-based algorithm and statistics-based algorithm that are able to jointly estimate the azimuth, elevation angles, and distance for the 3D positioning of the SRS transmitter (UE) are described in Section 3. The merits of the this work are summarized as follows:

- Minimum additional signaling and infrastructure: In this work, the positioning information is extracted by the propagation process estimation of the SRS on the receiver side. This means that the method can be applied to existing systems without additional signaling, protocol, or hardware/infrastructure modifications.
- Free from the multiple sites synchronization: Our work is different from TDoA-based multilateration or hyperbolic approaches that require costly timing synchronization between distributed sites and the positioning function. The joint azimuth, elevation, and delay estimation methods are used in this work.
- High capacity: Our work benefits from the orthogonality in the time and frequency domains of SRSs from the different UEs. The algorithms can be applied to each individual UE for positioning estimation without interference from other UEs. Theoretically, the positioning capacity equals the number of Zadoff–Chu sequences used for UEs.
- Flexibility: The position estimation algorithms of this work can easily adapt to the different subcarrier spacing and channel bandwidth combinations in the 5G NR for diverse positioning accuracy levels required by different application scenarios.

The rest of this paper is organized as follows: In Section 2, the system and signal models are describe to facilitate the discussion of the 3D positioning. Then, the subspace-based joint angle-delay estimation and EM-based signal clustering are introduced in Section 3. Section 4 shows the performances of both algorithms in different scenarios. The conclusion is given in Section 5.

## 2. System Hypotheses and Signal Modeling

### 2.1. Hypotheses

**Figure 1:**
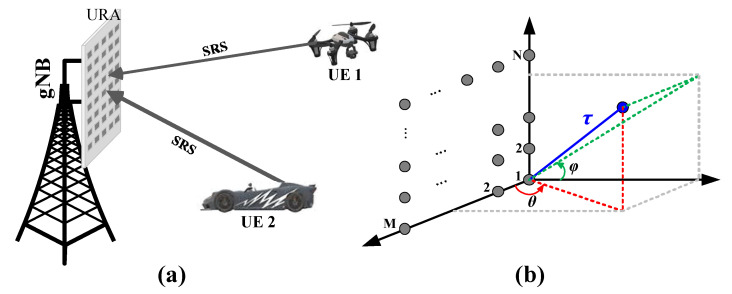
(**a**) System schematic diagram of the URA-based 5G NR 3D positioning; (**b**) URA geometry and dimension illustration. SRS, sounding reference signal.

In this paper, the main scope is to investigate the performance of 3D UE positioning rather than self-localization or navigation. The considered system sketch can seen in Figure 1a. To shed light on the positioning methods and performance metrics in the later sections, we list below the hypotheses and constraints at the beginning of the discussion:

- Frequency band: This work focuses on the sub-6 GHz band (e.g., 3.5 GHz) of the 5G NR carrier bands. Compared with the mmWave band, the sub-6GHz signal has less propagation loss and larger outdoor coverage, which is more suitable for manned or unmanned drones or vehicles.
- Receiving antenna: In order to obtain the 3D position of a signal source without trilateration or hyperbolic positioning, the positioning station (i.e., the base station) needs to be able to measure the azimuth, elevation angles, and the time delay simultaneously. Therefore, a uniform rectangular array (URA) is utilized at the receiver end to be capable of spanning the whole azimuth and elevation dimension. The modeling of URA is introduced in Section 2.3.
- Signal: The uplink 5G NR sounding reference signal (NR-SRS) sent by the UE is applied. The NR-SRS is an OFDM modulated Zadoff–Chu sequence that is feasible for time delay estimation. The introduction and modeling of SRS are given in Section 2.2.
- Algorithm: The extended subspace method and expectation-maximization (EM)-based algorithms are investigated for 3D positioning. The positioning algorithms are given in Section 3.
- Positioning host: The URA and positioning algorithms are hosted at the 5G base station (called gNB in 5G terminology), so as to leverage the computing power and energy supply to accommodate the large-scale URA in the sub-6 GHz band and run the positioning algorithms.

### 2.2. Sounding Reference Signal in 5G NR

**Figure 2:**
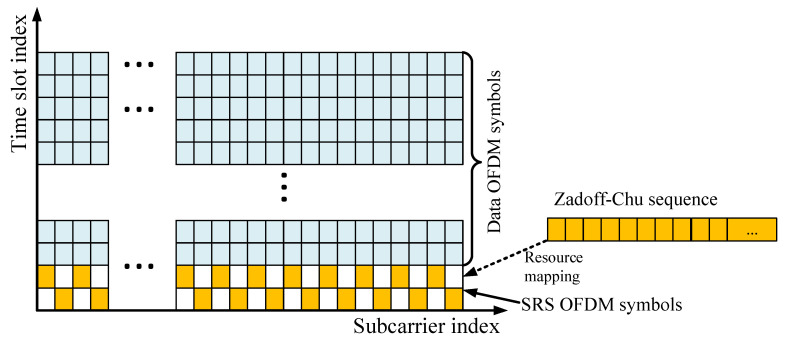
Simplified SRS generation: to map a Zadoff–Chu sequence (orange grids) to the specified subcarriers and time slots.

In 5G NR, the SRS is transmitted by the UE for uplink channel sounding, which includes the channel estimation (in the frequency domain) and synchronization. As defined in 3GPP TS 38.211 [16], an NR-SRS is an uplink orthogonal frequency division multiplexing (OFDM) signal filed with a Zadoff–Chu sequence on different subcarriers. For the purposes of communications, the SRS is used for closed-loop spatial multiplexing, uplink transmitting timing control, and reciprocity multi-user downlink precoding. To utilize the channel sounding function, the SRS must be known by both the UE (mobile transmitter) and gNB (base station receiver). With this prior knowledge known at the receiver, the SRS will be used to estimate the angle of the signal source and propagation delay by processing the received OFDM signal on an antenna array. In 5G NR, the SRS is transmitted as OFDM symbols, which are allocated in specified frequency (subcarrier) and time (slot) positions in 5G NR subframes. The generation of the SRS in 5G NR frames includes two steps: (i) Zadoff–Chu sequence $r_{\mathrm{zc}}\in C^{M_{sc,b}^{rs}\times 1}$ generation (described in Section 2.2.1); (ii) mapping $r_{\mathrm{zc}}$ to $s_{\mathrm{srs}}\in C^{W\times 1}$ as an OFDM symbol (described in Section 2.2.2). Figure 2 shows a simplified example of SRS generation.

#### 2.2.1. Zadoff–Chu Sequence Generation

Let us assume that a $s_{\mathrm{srs}}\in C^{W\times 1}$ is expected for a single-antenna UE. We start with the generation of Zadoff–Chu sequence $r_{\mathrm{zc}}\in C^{M_{sc,b}^{rs}\times 1}$ from Chapter 6.4.1.4.2 in the 3GPP standard TS 38.211 [16]. The length of $r_{\mathrm{zc}}$ is $M_{sc,b}^{rs}$, which is shorter than OFDM symbol *W*. $r_{\mathrm{srs}}$ is a variant of one of 30 base sequences r. We use u to indicate the base sequence index and v to denote different variants. The Zadoff–Chu sequence $r_{\mathrm{srs}}$ used for the 5G SRS can be obtained by using Equation (1):

(1) $$r_{\mathrm{zc}}\left(n,l^{{}^{\prime }}\right)=r_{u,v}\left(n\right),\;n=0,1,...,M_{sc,b}^{rs}-1;\;l^{{}^{\prime }}=0,1,...,N_{\mathrm{symb}}^{\mathrm{SRS}}-1$$

where $l^{{}^{\prime }}$ is the location index of the SRS OFDM symbol in a 5G subframe. $l^{{}^{\prime }}$ determines the variant index v (the detailed relation can be found in Appendix A.1). $l^{{}^{\prime }}$ can be chosen from zero to $\left(N_{\mathrm{symb}}^{\mathrm{SRS}}-1\right)$. $N_{\mathrm{symb}}^{\mathrm{SRS}}$ is received from the radio resource control (RRC) layer message, which indicates the maximum number of OFDM symbols that can be used for SRS transmissions in a 5G subframe. The length of $r_{\mathrm{srs}}$ is defined by the equation below:

(2) $$M_{sc,b}^{rs}=m_{\mathrm{SRS},b}N_{sc}^{RB}/K_{TC}$$

where $N_{sc}^{RB}$ is the number of subcarriers allocated for the Zadoff–Chu sequence element in each resource block (RB) and equal to 12 in this work, according to [10,16]. The allocated RB value of $m_{\mathrm{SRS},b}$ is chosen from a 64×4 SRS bandwidth configuration table defined in 6.4.1.4.3-1 [16], which is indexed by bandwidth configuration parameter $C_{SRS}$ and SRS transmission bandwidth indicator $B_{SRS}$, respectively. The value of structure controller parameter $K_{TC}$ can be chosen among 1, 2, and 4, and $\left(K_{TC}-1\right)$ indicates the number of empty subcarriers between two Zadoff–Chu elements in an SRS OFDM symbol.

#### 2.2.2. Resource Mapping

To transmit the SRS in the 5G NR frames, the generated Zadoff–Chu sequence $r_{\mathrm{zc}}\left(n,l^{{}^{\prime }}\right)$ in Section 2.2.1 is mapped to the given physical resources (which include subcarriers and time slots). The mapping can be described by Equation (3), which is defined in Chapter 6.4.1.4.3 of the 3GPP TS 38.211 specifications [16].

(3) $$S_{K_{\mathrm{TC}}k^{{}^{\prime }}+k_{0},\;l^{{}^{\prime }}+l_{0}}=\left\{\begin{array}{ccc} \beta_{\mathrm{SRS}}r_{\mathrm{zc}}(n,l^{{}^{\prime }}), & \;k^{{}^{\prime }}=0,1,\ldots ,M_{sc,b}^{RS}-1; & l^{{}^{\prime }}=0,1,\ldots ,N_{\mathrm{symb}}^{\mathrm{SRS}}-1\\ 0, & \;Otherwise \end{array}\right.$$

where subscripts $\left(K_{\mathrm{TC}}k^{{}^{\prime }}+k_{0}\right)$, $\left(l^{{}^{\prime }}+l_{0}\right)$ denote the subcarrier and time slot indices, respectively. $k_{0}$ and $l_{0}$ are the starting subcarrier index and starting time slot index. $\left(K_{\mathrm{TC}}k^{{}^{\prime }}+k_{0}\right)$ and $l^{{}^{\prime }}$ are the shift from the starting position in the frequency domain and the time domain, respectively. $N_{\mathrm{symb}}^{\mathrm{SRS}}$ and $M_{sc,b}^{RS}$ are explained in Section 2.2.1. The parameter $\beta_{\mathrm{SRS}}$ is the power constraint of the SRS specified in [10]. It ensures that the total uplink power of UEs is controlled under the same standards. The mapping rules are described in Appendix A.2. After the resource mapping, the the original Zadoff–Chu sequence $r_{\mathrm{zc}}\in C^{M_{sc,b}^{Rs}\times 1}$ is arranged into specific subcarrier and time slots to form the SRS OFDM symbol $s_{\mathrm{srs}}\in C^{W\times 1}$ ($s_{\mathrm{srs}}$ is the transpose of one column of $S_{K_{\mathrm{TC}}k^{{}^{\prime }}+k_{0},\;l^{{}^{\prime }}+l_{0}}$). $s_{\mathrm{srs}}$ is then modulated onto the OFDM subcarriers:

(4) $$s=s_{\mathrm{srs}}\cdot \left[1,e^{-2j\pi f_{1}t_{0}},e^{-2j\pi f_{2}t_{0}},...,e^{-2j\pi f_{W-1}t_{0}}\right]$$

where the operator (·) is the point product and $f_{w}=\left(w-1\right)\cdot \Delta f$ (Δf is the OFDM subcarrier spacing).

#### 2.2.3. Propagation Delay Impact on SRS OFDM Symbols

The SRS OFDM symbol s is transmitted from the UE to gNB. Assuming the propagation causes the delay τ of the signal, the delay τ will introduce the different phase shift $\gamma_{w}=e^{-j2\pi f_{w}\tau }$ on the $w_{th}$ subcarrier. Thus, the time delayed version s can be written as $s\left(\tau \right)=s_{\mathrm{srs}}\cdot \left[1,e^{-2j\pi f_{1}\left(t_{o}+\tau \right)},e^{-2j\pi f_{2}\left(t_{o}+\tau \right)},...,e^{-2j\pi f_{W-1}\left(t_{o}+\tau \right)}\right]$. We define the delay manifold as:

(5) $$g\left(\tau \right)={\left[1,e^{-2j\pi f_{1}\tau },e^{-2j\pi f_{2}\tau },...,e^{-2j\pi f_{W-1}\tau }\right]}^{T}$$

Zadoff–Chu sequences and resource mapping rules are designated by gNB to the UE. In the 5G NR system, both the transmitter (UE) and receiver (gNB) have prior knowledge of the SRS OFDM symbol. The manifold g(τ) can be used to estimate the propagation delay between the UE and gNB, which is described in Section 3.1 and Section 3.2.

### 2.3. Uniform Rectangular Array Signal Model

The URA with M×N elements is illustrated in Figure 1b. *M* and *N* denote the number of elements on the x-axis and z-axis, respectively. The antenna elements are half-wavelength spaced horizontally and vertically. We use symbols θ and ϕ to denote the azimuth and elevation angle, respectively, and use the element at the original point as the reference element. We use an incident signal of a single source k from direction $\left(\theta_{k},\phi_{k}\right)$. The array manifold vector can be written as:

(6) $$a\left(\theta_{k},\phi_{k}\right)=a_{z}\left(\theta_{k}\right)⊗a_{e}\left(\phi_{k}\right)$$

where the operator ⊗ is the Kronecker product. $a_{z}\left(\theta_{k}\right)$ and $a_{e}\left(\phi_{k}\right)$ are the azimuth and elevation manifolds, respectively, defined as:

(7a) $$\begin{array}{cc} \; & a_{z}\left(\theta_{k}\right)=e^{\frac{-j2\pi d}{\lambda }}{\left[1,e^{-j\pi \lambda cos\theta_{k}},...,e^{-j\left(M-1\right)\pi \lambda cos\theta_{k}}\right]}^{T} \end{array}$$

(7b) $$\begin{array}{cc} \; & a_{e}\left(\phi_{k}\right)=e^{\frac{-j2\pi d}{\lambda }}{\left[1,e^{-j\pi \lambda cos\phi_{k}},...,e^{-j\left(N-1\right)\pi \lambda cos\phi_{k}}\right]}^{T} \end{array}$$

where λ is the wavelength and *d* is the array element spacing, which is the half wavelength in this work. For a single snapshot (sample), the congregate receiving signal from K sources can be written as:

(8) y=A(θ,ϕ)x+n

where $A\left(\theta ,\phi \right)=\left[a\left(\theta_{1},\phi_{1}\right);a\left(\theta_{2},\phi_{2}\right);...,a\left(\theta_{K},\phi_{K}\right)\right]\in C^{MN\times K}$, $y\in C^{MN\times 1}$, $x\in C^{K\times 1}$, and $n\in C^{MN\times 1}$ are one snapshot (sample) of the array receiving signal, incident signal, and noise, respectively.

### 2.4. Signal Model for 3D Positioning

In Equation (8), the signal x is only one snapshot from the K signal sources. Furthermore, the receiving signal y only contains the azimuth and elevation angles. In order to integrate the delay information contained in the SRS for 3D positioning (Section 2.2.3), the $x\in C^{K\times 1}$ in Equation (8) is replaced by the multipath propagated SRS $S\left(\tau \right)\in C^{K\times W}$. *W* is the length of the SRS. $S={\left[s_{1},s_{2},...,s_{k}\right]}^{T}$. Then, Equation (8) can be rewritten as:

(9) Y=A(θ,ϕ)S(τ)+N

where $Y\in C^{MN\times W}$, and $N\in C^{MN\times W}$ are the receiving SRS and noise, respectively, $\tau =[\tau_{1},\tau_{2},...,\tau_{k}]$. Y contains the azimuth, elevation angles, and delay information. To facilitate the 3D positioning, we further define the 3D manifold vector:

(10) u(θ,ϕ,τ)=A(θ,ϕ)⊗g(τ)

where $u\left(\theta ,\phi ,\tau \right)\in C^{MNL\times 1}$ is the 3D manifold vector. Similarly, the receiving signal Y is vectorized to Y=vec(Y).

## 3. 3D Positioning Algorithms

### 3.1. Subspace-Based Approach

The subspace-based signal classification is a widely used approach for angle estimation of the signal source. Most of the works can be traced back to the multiple signal classification (MUSIC) algorithm [17,18]. a similar approach is also applied on the multiple carrier signal for signal delay measurement [19]. The subspace approach is also used for joint 2D delay-angle estimation of the radio source be using the spatial-time manifold [20,21]. In this paper, we extend the manifold to the 3D spatial-time searching space u(θ,ϕ,τ), as shown in Equation (10). To performance the 3D position estimation, we first calculate the auto-correlation matrix:

(11) $$R_{\mathrm{YY}}=E\left[YY^{*}\right]$$

where E[·] denotes expectation and $Y^{*}$ is the conjugate transpose of Y. We take the eigenvalue decomposition of $R_{\mathrm{YY}}$ to obtain the eigenvalue vector $\lambda =[\lambda_{1},\lambda_{2},...,\lambda_{MNL}]$ and eigenvector matrix $e=[e_{1},e_{2},...,e_{MNL}]$. The eigenvalues $\lambda_{i}$ in λ are in ascending order, and $e_{i}$ corresponds to $\lambda_{i}$. Assume there are K sources. We can define the noise subspace as Equation (12):

(12) $$E_{n}=\left[e_{1},e_{2},...e_{MN-(K+1)}\right]$$

With the noise subspace, the 3D angle-time spectrum can be defined as:

(13) $$P\left(\theta ,\phi ,\tau \right)=\frac{u^{*}\left(\theta ,\phi ,\tau \right)u\left(\theta ,\phi ,\tau \right)}{u^{*}\left(\theta ,\phi ,\tau \right)E_{n}E_{n}^{*}u\left(\theta ,\phi ,\tau \right)}$$

The azimuth angle θ, elevation angle ϕ, and time delay τ that are associated with the peak values in the 3D space P(θ,ϕ,τ) determine the estimated signal source position.

### 3.2. Statistics-Based Approach

**Figure 3:**
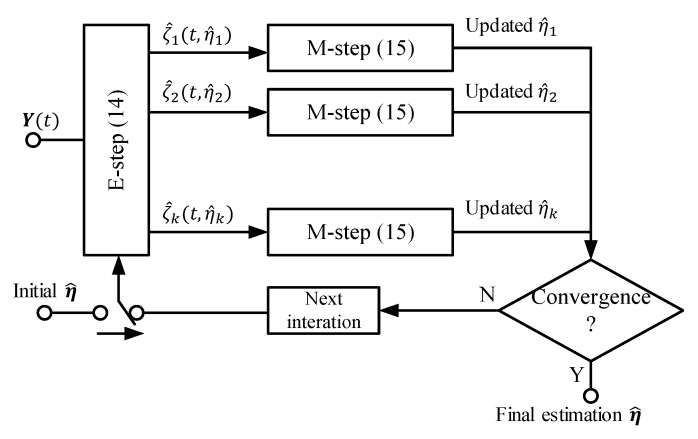
The signal flow of the EM algorithm. This processing flow was also used in [23].

Another method used in this work is space-alternating generalized expectation-maximization (SAGE) [22,23], which is based on the EM [24] algorithm. The EM algorithm is used to estimate latent states or parameters when parts of the observations are missing or censored. In general, it is an integration process that contains an expectation step (E-step) and a maximization step (M-step). In the context of the statistics approach, we can define the vector $\eta_{k}=\left[\theta_{k},\phi_{k},\tau_{k}\right]$ to indicate the position of the $k_{th}$ source. In the E-step, the estimate of the $k_{th}$ source ${\hat{x}}_{k}\left(t;\hat{\eta }\right)$ of the current iteration can be written as:

(14) $${\hat{x}}_{k}\left(t;\hat{\eta }\right)=\zeta_{k}\left(t;\hat{\eta }\right)+\beta_{k}N_{k}\left(t\right)$$

where $\zeta_{k}\left(t;\hat{\eta }\right)=a\left(\theta_{k},\phi_{k}\right)s_{k}$ is the assumed receiving signal from the $k_{th}$ source on the URA without noise, $\beta_{k}$ are non-negative parameters, $\sum_{k=1}^{K}\beta_{k}=1$ holds, and $N_{k}\left(t\right)=Y\left(t\right)-\sum_{k=1}^{K}\zeta_{k}\left(t;\hat{\eta }\right)$ is intermediate noise. In the M-step, the updated value of $\eta_{k}=\left[\theta_{k},\phi_{k},\tau_{k}\right]$ can be obtained:

(15) $${\hat{\eta }}^{\prime }\left({\hat{x}}_{k}\right)=\arg\max_{\left[\theta_{k},\phi_{k},\tau_{k}\right]}\int_{0}^{T}a^{*}\left(\theta ,\phi \right){\hat{x}}_{k}\left(t;\hat{\eta }\right)s^{*}\left(t-\tau \right)dt$$

where $a\left(\theta ,\phi \right)\in C^{1\times MN}$ is the URA manifold and *T* is the observing duration, which is selected to cover the sequence length and maximum propagation delay. The value of *T* in this work equals one slot time. s(t) is the ideal original SRS. The E-step and M-step are iteratively implemented until the algorithm reaches the convergence point. In this paper, we use the value of intermediate noise $N_{k}\left(t\right)$ as the convergence condition. The value of $N_{k}\left(t\right)$ keeps reducing in each EM iteration and reaches its extreme limit point when the estimated parameters are approximately fully recovered. The extreme limit point of $N_{k}\left(t\right)$ is reached when the power difference of $N_{k}^{step(n)}\left(t\right)$ and $N_{k}^{step(n-1)}\left(t\right)$ approaches zero. The flowchart of the algorithm is shown in Figure 3:

## 4. Performance Comparison

### 4.1. Simulation Setting and Examples

#### 4.1.1. Simulation Setting

The positioning performance evaluation was carried out using simulations and the system parameters. The SRS configuration and positioning algorithm parameters are listed in Table 1, Table 2 and Table 3.

#### 4.1.2. Example SRS and Positioning Result

**Figure 4:**
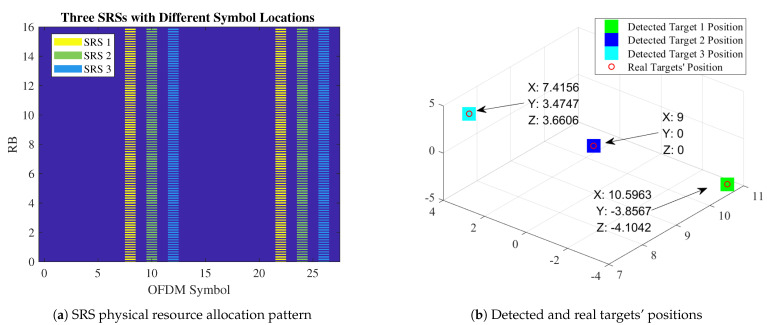
Positioning accuracy of the EM and subspace algorithms in different SNR conditions.

In this section, the example SRS used in the simulation in Section 4.2 for the positioning of three different UEs is shown in Figure 4a,b.

### 4.2. Performance Comparison of Positioning Accuracy in Different Contexts

In this section, we use the RMSE as the metric for the estimation accuracy of the EM and subspace algorithms. The varying parameters in the following subsections are the SNR, antenna scale, and subcarrier space. Due to the fact that SRSs from different UEs are orthogonal in the time, frequency, and codes domain, the positioning of multiple UEs can be directly decomposed into single target positioning. Therefore, the simulation results shown in this section were executed as single target detection.

#### 4.2.1. Single Target Estimation Performance

**Figure 5:**
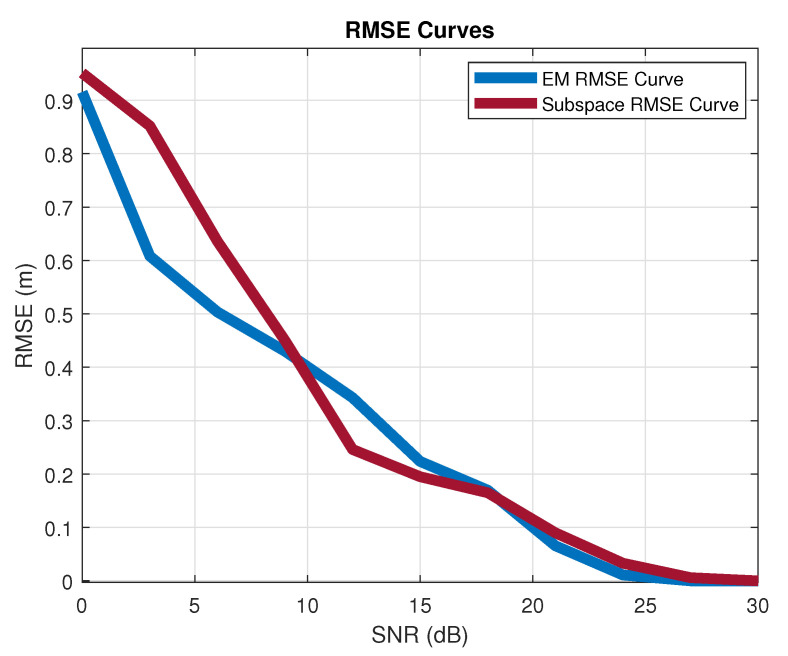
Positioning accuracy of the EM and subspace algorithms in different SNR conditions.

Firstly, Figure 5 is given to show how the level of SNR affects the estimation accuracy. The target is 3 m away from the base station, and the SRS is from the direction of [20,20] in the azimuth and elevation dimension. The bandwidth assigned for the SRS is 2.88 MHz, which contains 192.15 kHz subcarriers with a comb-two structure. Ninety-six subcarriers contain the SRS message. The size of the URA for AoA estimation is 8×8. Both the EM and subspace methods provide accurate estimation in the high SNR region (>10 dB), and the EM algorithm shows better robustness in the low SNR region (0∼10 dB). The reason for this phenomenon is that the iteration procedures (especially the M-step) in the EM algorithm effectively approach the maximum likelihood estimation.

**Figure 6:**
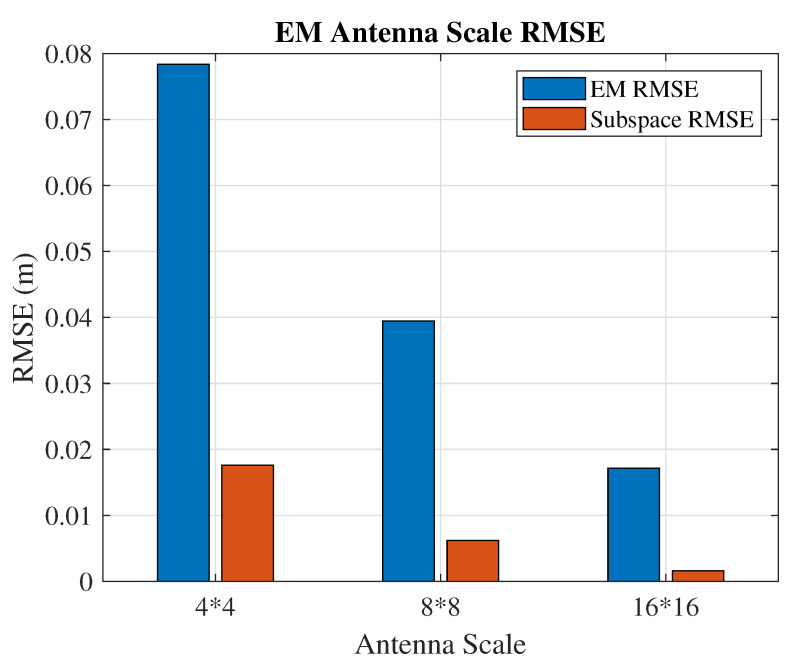
Positioning accuracy of the EM and subspace algorithms with known distance and estimated AoA.

This 3D positioning accuracy is affected by the antenna scales and signal bandwidth. To better understand the antenna scale effect on the RMSE in both the EM and subspace approaches, Figure 6 shows the comparisons under different antenna scales. The signal bandwidth is 2.88 MHz, and the SNR is 9 dB. To achieve better angle estimation accuracy, zero-point-zero-five degrees are set as the angle increment size. With the fixed target position in both cases, the RMSEs of the position estimation decrease with the larger antenna scale. In this comparison, we assume both algorithms know the signal source distance and perform the angle-only estimation. It is evident that the subspace algorithm has the advantage on AoA recognition.

**Figure 7:**
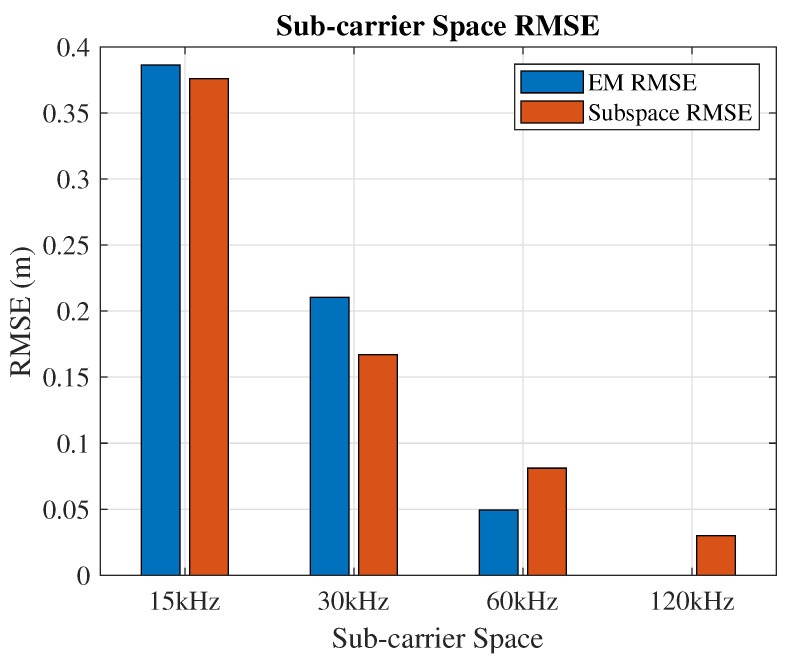
Positioning accuracy of the EM and subspace algorithms with different bandwidths.

ToA accuracy is related to the subcarrier space, while the number of SRS subcarriers is fixed at 96. In 5G NR, subcarrier spacing can be chosen from 15, 30, 60, and 120 kHz. An 8×8 URA, 1 ns time step, and 9 dB SNR are used to figure out the subcarrier spacing impacts. Different subcarrier spacings will lead to different signal bandwidths. For the results shown in Figure 7, the performance of the 8×8 antenna array is shown under the 9 dB SNR. With the increasing of the subcarrier spacing, both the EM and subspace algorithms show improving positioning resolution. The EM algorithm outperforms the subspace algorithm in the larger subcarrier spacing cases (60 and 120 kHz), while the subspace algorithm outperforms the EM algorithm in the narrower subcarrier spacing cases (15 and 30 kHz).

#### 4.2.2. Impact of the Nearby Reflection

This section discusses the deterioration caused by a nearby reflected signal. Different from the SRSs from the different UEs, the reflection of the SRS from a nearby object has a limited ToA difference from the original SRS. In this simulation, we placed the signal source and reflection point within the minimal resolvable area ($1.5^{\circ }$ and 0.3 m target range in the simulation when 8×8 is the antenna scale). Assume the signal source is located at a fixed point with a 12 m distance to the base station with [20,20] azimuth and elevation angles, under 9 dB SNR. The reflecting signal is located at 12.6 m with [21,21] azimuth and elevation angles. Under that case, the ToA detection for both the EM and subspace approaches is influenced. The angle and time increment are one degree and 1 ns, respectively. The details are discussed in the following subsection.

**Figure 8:**
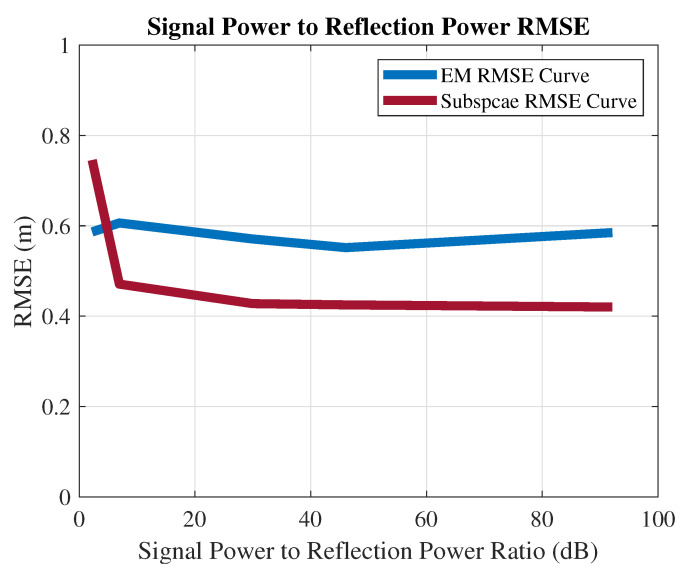
The impact of a nearby coherent source on the positioning accuracy.

RMSE vs. power: The total bandwidth is 60 MHz, and the SRS uses 2.88 MHz. The reflection signal is set at a fixed range 12.6 m and the [21,21] angles. The EM and subspace algorithms’ positioning RMSEs versus different signal power-to-reflection power ratio (SPRP) levels are collected in Figure 8. The EM estimation accuracy decreases with the power of the reflection. However, the influence of the SPRP on the subspace algorithm is more severe (constantly around 0.6, as shown in Figure 5, as the subspace algorithm is more sensitive to the correlated sources.

### 4.3. Limitations and Discussion

From the simulation results shown in Section 4.2, we can see that both the subspace and EM algorithms can successfully localize the SRS source by estimating the angle and delay. The EM algorithm outperforms the subspace algorithm in terms of the position RMSE. However, the current methods have their limitations, which need to be addressed in future work:

- Computational load of the subspace algorithm: As can be seen from Equations (6), (10), and (11), the subspace algorithm involves two Kronecker operations and one covariance operation of the Kronecker product. These facts imply that the vector and matrix size will increase exponentially with the number of antenna elements and the subcarriers; moreover, the base station will have to allocate more computing resources for positioning information extraction. Therefore, the development of a computational efficient subspace-based algorithm will pave the wary for applying the subspace-based algorithms in practice.
- Multipath propagation: The orthogonality of the SRSs from different UEs avoids the mutual interference to achieve the high positioning capacity. However, the multipath distortions of the ToA parameter estimation is unavoidable because the multiple copies of the signal originated from the same SRS with a recognizable ToA difference are strongly correlated. As can be seen from the results in Section 4.2.2, the estimation RMSE of the position increases if there exists a copy of the SRS close to the UE. Hence, the multipath mitigation schemes with high spatial resolution are the problem to be solved in future work.
- UEs’ mobility: The mobility of the UEs will cause position changes during the 20 ms observation time of the SRS in this work. By using the same codes that generated the results in Section 4.2, we find that the estimated position of a moving UE is located at the middle point between the two positions of that UE when the observation starts (0 ms) and ends (20 ms). For example, let us assume that the mobility of one target is 70 km/h. The radial distance between the positions when the SRS observation started at (0 ms) and ended at (20 ms) is then 0.39 m. Under no reflections and a line-of-sight scenario, the estimated position radial distance to the UE’s final position is 0.19 m (around 0.39/2). This example shows that mobility related error exists, and it increases with UE speed. In order to reduce the performance reduction introduced by the mobility of UEs, reducing the observation time can be a straightforward approach.
- Non-line-of-sight positioning: Regarding the non-line-of-sight scenario, this work mainly has two issues that are addressed. The first one is related to the multipath propagation. Multipath indeed makes the estimation difficult as we discussed in the multipath part, when we showed how the nearby reflected signal decreased the estimation accuracy. Furthermore, the position estimation in this case is more challenging than for the line-of-sight, as it has a weak signal strength and a small ToA difference. The second issue is related to the fact that this design can directly estimate the incident angle rather than the reflection angle on the surface where reflection and scattering occur. Thus, this design shall utilize the environment reflection information and the UE’s SRS to jointly calculate the target’s position.
- Requirement of prior knowledge: Both the subspace and EM algorithms in this paper take advantage of the prior knowledge of the SRS and channel equalization to estimate the angle and propagation delay. However, the SRS is only sparsely distributed in the 5G NR frames. These facts limited the effective samples that can be used for positioning purposes. In addition, both algorithms need to know the number of signal sources to achieve good estimation accuracy. Thus, the blind source separation or blind estimation schemes are also promising topics to explore in the context of 5G NR signal-based positioning.

## 5. Conclusions

In this paper, the subspace- and EM-based signal station 3D UE positioning methods in the 5G network are proposed. The positioning function is facilitated by the uplink SRS emitted by the UE and by the antenna array-equipped 5G base station. The 3D positioning performances of both algorithms are investigated under various SNRs, array configurations, channel bandwidths, and multipath scenarios. The simulation results show that both the subspace- and EM-based methods are able to accurately estimate the azimuth/elevation angles and time delay with the 3D signal manifold. We also note that the EM-based method outperforms the subspace-based method in the low SNR region in both the estimation error and resolution. Moreover, the EM-based approach presents the advantage of a lower computational resource cost than the subspace-based approach. Both proposed methods show the capability of achieving different positioning resolutions and accuracy levels by using a flexible signal bandwidth and subcarrier spacing, which is provided by the 5G NR for the different application scenarios. In addition, the orthogonality of the SRSs from different UEs provides excellent conditions for both positioning approaches to detect, localize, and keep tracking a large number of UEs without mutual interference and performance loss. However, the multipath propagation, and especially the reflections close to the UEs, will deteriorate both the accuracy and the resolution performance. Therefore, our future work will focus on the environment-robust 3D positioning method in 5G networks, aiming at better multipath mitigation and better dealing with non-line-of-sight scenarios.

## Figures and Tables

**Table 1:** System parameters.

Carrier frequency	The n78 3.5 GHz in Frequency Range 1 (FR1, sub-6 GHz) frequency band, the most common 5G band in deployed networks. Furthermore, this band is more suitable for the outdoor scenarios concerned in this work.
Receiving array	Square shaped, half wavelength spacing URA is used. The 4×4, 8×8, and 16×16 configurations are used to test the array scale impact on the angle estimation performance.
Bandwidth	Though the supported channel bandwidth of n78 band is from 10 to 100 MHz, different values are used in this paper to test the bandwidth impact on the delay estimation performance.
Sub-carrier interval	The default subcarrier spaces 15 kHz, 30 kHz, 60 kHz, and 120 kHz are used to test the bandwidth impact on the positioning performance.
Modulation	The SRS is an OFDM signal. However, it is not a data payload. Thus, the symbols allocated to OFDM subcarriers are not Zadoff–Chu code elements, which do not have any modulation.
Channel model	The additive white Gaussian noise (AWGN) complex channel is used.
Duplex mode	Time-division duplexing (TDD) is the most used duplex mode in the n78 band. The positioning methods in this work are neutral to the duplex mode.

**Table 2:** SRS configuration. RB, resource block; RRC, radio resource control.

$C_{srs}$	42	The total bandwidth configuration index to the maximum available RBs can be used.
$B_{srs}$	2	The transmission bandwidth selecting index, used with $C_{srs}$ for selecting the RBs for the SRS.
$K_{TC}$	2	The comb structure, which indicates the number of subcarrier gaps between two SRS subcarriers.
$l_{0}$	0	The symbols’ start position index carried in the RRC message. Controls the SRS starting position range from the 8th to 13th OFDM symbol.
$k_{0}$	0	The SRS subcarrier starting position. Frequency hopping and sequence hopping are disabled in the simulations. The subcarrier mapping starts from the first subcarrier.

**Table 3:** Positioning algorithms’ parameters.

Angle step	0.1 degrees	The AoA searching step in the EM and subspace-based estimation process.
Time step	1 ns	The ToA searching step.
Observation time	20 ms	Observation time set as 20 ms to enhance the performance and that equals 20 time slots when the subcarrier spacing is 15 kHz.
Sampling frequency	60 MHz	Assuming a 60 MHz bandwidth. Sixteen RBs are allocated to the UEs, and the sampling frequency can be selected as 60 Mhz.

## Appendix A. Zadoff–Chu Base Sequence and Resource Mapping Rules

### Appendix A.1. Zadoff–Chu Base Sequence and Variants

If $m_{\mathrm{SRS},b}/K_{\mathrm{TC}}<6$, the sequence group is generated from one base sequence with v = 0. Once the value of $m_{\mathrm{SRS},b}/K_{\mathrm{TC}}⩾6$, the value of v is chosen from zero and one according to Equation (A3). Basically, the values of sequence group u are set for enabling group hopping, and sequence number v is the set ofsequence hopping functions. u can be calculated by Equation (A1a).

(A1a) $$\begin{array}{cc} u & =(f_{\mathrm{gh}}+n_{ID}^{SRS})\;mod\;30 \end{array}$$

(A1b) $$\begin{array}{cc} f_{\mathrm{gh}} & =\sum_{m=0}^{7}c\left(8(n_{s,f}^{\mu }N_{\mathrm{symb}}^{\mathrm{slot}}+l_{0}+l^{{}^{\prime }})+m\right)2^{m}\;mod\;30 \end{array}$$

where $n_{ID}^{SRS}$ is the SRS sequence identity received from the RRC message, $n_{s,f}^{\mu }$ is the slot number within a frame for the subcarrier spacing configuration, $N_{\mathrm{symb}}^{\mathrm{slot}}$ is the mean number of OFDM symbols per slot, and $l_{0}$ and $l^{{}^{\prime }}$ indicate the starting location of the SRS OFDM symbols and the location of the $l^{{}^{\prime }}$th symbol. $f_{\mathrm{gh}}$ can be calculated from Equation (A1b) when RRC parameter “groupOrSequenceHopping” contains information “groupHopping”; otherwise, it equals zero. c(n) is the PNsequence given by [16] as shown by the equation group (A2):

(A2a) $$\begin{array}{cc} c(n) & =(x1(n+1600)+x2(n+1600))\;mod\;2 \end{array}$$

(A2b) $$\begin{array}{cc} x1(n+31) & =(x1(n+3)+x1(n))\;mod\;2,\;(\mathrm{initial}\;[x1(0)=1,x1(n)=0]) \end{array}$$

(A2c) $$\begin{array}{cc} x1(n+31) & =\left(x1\left(n+3\right)+n+2\right)+n+1)+n))\;mod\;2,\;\left(\mathrm{initial}\;\left[n_{ID}^{SRS}=\sum_{i=0}^{30}\left(x_{2}\left(i\right)2^{i}\right]\right)\right) \end{array}$$

If the RRC parameter “groupOrSequenceHopping” contains information “sequenceHopping” and sequence length $M_{sc,b}^{rs}$ is not less than 6 RB in length ($6N_{sc}^{RB}$), v can be calculated by Equation (A3):

(A3) $$v=c(n_{s,f}^{\mu }N_{\mathrm{symb}}^{\mathrm{slot}}+l_{0}+l^{{}^{\prime }})$$

otherwise, v equals zero.

### Appendix A.2. Sub-Carrier and Time-Slot Mapping

This Appendix introduces the resource-mapping method including determining the starting time slot index $l_{0}$, starting subcarrier index $k_{0}$, and mapping rules.

The starting time slot index $l_{0}$ is derived from Equation (A4) and two parameters $N_{symb}^{slot}$ and symbol offset $l_{offset}$, which are contained in the “resourceMapping” of the RRC message.

(A4) $$l_{0}=N_{symb}^{slot}-1-l_{offset}$$

The starting subcarrier index $k_{0}$ is derived from Equation (A5a):

(A5a) $$\begin{array}{cc} k_{0} & =\bar{k_{0}}+\sum_{b=0}^{B_{SRS}}K_{\mathrm{TC}}M_{sc,b}^{SRS}n_{b} \end{array}$$

(A5b) $$\begin{array}{cc} \bar{k_{0}} & =n_{shift}N_{sc}^{RB}+K_{TC} \end{array}$$

We can see that comb structure parameter $K_{TC}$, $M_{sc,b}^{SRS}$ and SRS transmission bandwidth indicator $B_{SRS}$ directly influence the starting position $k_{0}$. $n_{shift}$ is the parameter contained in the RRC message subsection “freqDomainShift” for adjusting the SRS allocation to align with the common resource block grid in multiples of four.

In order to change the SRS physical resource allocation pattern with different time slots, frequency hopping is used by controlling the frequency domain index $n_{b}$ to further change the starting subcarrier index $k_{0}$. $b_{hop}$ and $B_{SRS}$ are frequency hopping function controlling the parameters, which are carried in the RRC message. The frequency position index $n_{b}$ in Equation (A5a) has different values depending on the frequency hopping function’s on/off status, as shown in Equation (A6).

(A6) $$n_{b}=\left\{\begin{array}{ccc} 4n_{RRC}/m_{SRS,b}\;mod\;N_{b}, & \mathrm{If} & \;b_{hop}\geq B_{SRS}\\ \left(4n_{RRC}/m_{SRS,b}\right)\;mod\;N_{b}, & \mathrm{If} & \;b_{hop}<B_{SRS}\;\&\;b<b_{hop}\\ F_{b}\left(n_{SRS}\right)+4n_{RRC}/m_{SRS,b}\;mod\;N_{b}, & \mathrm{If} & \;b_{hop}<B_{SRS}\;\&\;b\geq b_{hop} \end{array}\right.$$

When $b_{hop}$ is bigger than $B_{SRS}$, the frequency hopping is disabled, and $n_{b}$ will have a constant value, as shown in the first case of Equation (A6), unless the SRS configuration is reset. Once $b_{hop}$ is smaller than $B_{SRS}$, frequency hopping is enabled, and $n_{b}$ will be calculated according to the second and third cases in Equation (A6). The $F_{b}$ in the third case of Equation (A6) is defined in Equation (A7), which does not have a specific physical meaning, but is a parameter used in the calculation for simplicity. Furthermore, the quantity $n_{RRC}$, ranging from zero to 67, is given by the RRC message parameter “freqDomainPosition”, which is used for modifying the SRS sequence frequency domain position. It should be mentioned that the value of *b* in Equation (A7) is equal to $N_{b}$.

(A7) $$F_{b}=\left\{\begin{array}{cccc} \; & \left(N_{b}\right)/2)\left[\frac{n_{SRS}\;mod\;\prod_{b^{\prime }=b_{hop}}^{b}N_{b^{\prime }}}{\prod_{b^{\prime }=b_{hop}}^{b}N_{b^{\prime }}}\right]+\left[\frac{n_{SRS}\;mod\;\prod_{b^{\prime }=b_{hop}}^{b}N_{b^{\prime }}}{2\prod_{b^{\prime }=b_{hop}}^{b}N_{b^{\prime }}}\right], & \mathrm{If} & \;N_{b}\;\mathrm{is}\;\mathrm{even}\\ \; & \left(N_{b}\right)/2)\left[n_{SRS}\;mod\;\prod_{b^{\prime }=b_{hop}}^{b}N_{b^{\prime }}\right], & \mathrm{If} & \;N_{b}\;\mathrm{is}\;\mathrm{odd} \end{array}\right.$$

All in all, the Zadoff–Chu sequences are arranged into specific subcarriers and time slots to form the SRS OFDM symbols with the rules defined in the standard; the final SRS information carrying OFDM signal $S_{\mathrm{srs}}\in C^{N_{sc}^{RB}N_{RB,UL}\times W}$ contains the total SRS sequence $r_{zc}\in C^{M_{sc,b}^{SRS}\times N_{\mathrm{symb}}^{\mathrm{SRS}}}$. The $N_{RB,UL}$ means the number of RBs allocated to the UEs, and the value depends on the UEs’ total bandwidth and subcarrier spacing. Although it varies from case to case, 3GPP [25] has defined the maximum and minimum values of $N_{RB,UL}$ to be 20 and 275, while the subcarrier spacing is less than 240 kHz.
